# Viewpoint on WHO implementation guidance on tuberculosis infection prevention and control

**DOI:** 10.1183/13993003.00109-2024

**Published:** 2024-12-12

**Authors:** Onno W. Akkerman, Giovanni Battista Migliori, Dennis Falzon, Alberto L. Garcia-Basteiro, Avinash Kanchar, Olha Konstantynovska, Fusun Oner Eyuboglu, Raquel Duarte

**Affiliations:** 1 University of Groningen, University Medical Center Groningen, Department of Pulmonary Diseases and Tuberculosis, Groningen, The Netherlands; 2 University of Groningen, University Medical Center Groningen, TB Center Beatrixoord, Groningen, The Netherlands; 3 Servizio di Epidemiologia Clinica delle Malattie Respiratorie, Istituti Clinici Scientifici Maugeri IRCCS, Varese, Italy; 4 World Health Organization, Global Tuberculosis Programme, Geneva, Switzerland; 5 Centro de Investigação em Saúde de Manhiça (CISM), Maputo, Moçambique; 6 Instituto de Salud Global de Barcelona (ISGlobal), Hospital Clínic-Universitat de Barcelona, Barcelona, Spain; 7 Centro de Investigación Biomédica en Red de Enfermedades Infecciosas (CIBERINFEC), Barcelona, Spain; 8 V.N. Karazin Kharkiv National University, Department of Infectious Diseases and Clinical Immunology, Kharkiv, Ukraine; 9 FOE Respiratory Clinic, Ankara, Turkey; 10 Baskent University Division of Pulmonary Diseases, Ankara, Turkey; 11 EPIUnit – Instituto de Saúde Pública, Universidade do Porto, Porto, Portugal; 12 Laboratório para a Investigação Integrativa e Translacional em Saúde Populacional (ITR), Porto, Portugal; 13 ICBAS – Instituto de Ciências Biomédicas Abel Salazar, Universidade do Porto, Porto, Portugal; 14 Instituto de Saúde Pública Doutor Ricardo Jorge (INSA Porto), Porto, Portugal; 15 Contributed equally

## Abstract

Transmission continues to drive the tuberculosis (TB) and drug-resistant TB epidemics, making infection control an essential component for public health agencies worldwide [1–3]. Transmission of TB is complex, influenced by factors linked to patient behaviour, the form of disease, the exposed individual, the microbe and the environment [3–6]. Each year, more than 10 million people develop TB, more than 80% of whom have pulmonary disease and more than 60% are bacteriologically positive [1].

## Shareable abstract

**This viewpoint describes key aspects for TB infection prevention and control, and also covers areas of paramount interest for clinicians, such as when to admit or discharge infectious patients and presenting relevant country examples** https://bit.ly/3YcyJxj

## Introduction

Transmission continues to drive the tuberculosis (TB) and drug-resistant TB epidemics, making infection control an essential component for public health agencies worldwide [1–3]. Transmission of TB is complex, influenced by factors linked to patient behaviour, the form of disease, the exposed individual, the microbe and the environment [3–6]. Each year, more than 10 million people develop TB, more than 80% of whom have pulmonary disease and more than 60% are bacteriologically positive [1]. Determining infectiousness is difficult in the absence of a reliable biomarker for point-of-care triage of TB patients. While sputum bacteriology status correlates with infectiousness, its accuracy is limited, especially after initiating effective TB treatment [3, 7].

Although TB can affect anyone, certain groups are at higher risk of infection or disease progression. These high-risk groups include individuals with HIV, those in contact with TB patients (including healthcare workers), children and people in crowded settings, such as prisons, correctional centres, refugee camps and elder care homes.

The challenges and costs of managing TB highlight the need to prevent *Mycobacterium tuberculosis* transmission, especially in healthcare facilities and congregate settings. As part of its three-pillar approach, the World Health Organization (WHO)’s End TB Strategy underlines strengthening TB prevention, focusing on TB infection prevention and control (TB-IPC) in high-risk areas.

This article explores the WHO's recent TB-IPC operational handbook [8], its potential impact and its challenges in implementation, and country-specific experiences with TB-IPC.

## Highlights from the WHO guidelines and handbook

The WHO's TB-IPC guidance outlines a three-tier hierarchy for TB-IPC, which includes administrative controls, environmental controls and respiratory protection. The guidance highlights the importance of quick identification, triage, separation and treatment of TB cases to reduce the release of infectious particles into the air and to minimise the exposure to persons with infectious TB [3, 8]. These administrative control measures form the backbone of the FAST approach (Find cases Actively, Separate safely, Treat effectively) [3, 8]. In addition, infectious individuals should be encouraged to observe respiratory hygiene in all settings. Ventilation plays an important role in TB-IPC, to reduce *M. tuberculosis* transmission. It is considered a core component of environmental control. Natural ventilation uses doors and windows, mechanical ventilation offers controlled air changes using air-conditioning equipment and filtration, and “mixed-mode” solutions utilise both [3, 8]. Upper room germicidal ultraviolet (GUV) systems can complement other environmental control measures, but should not replace them [3, 8]. Respiratory protection is the final component of TB-IPC. Respirators are recommended to protect healthcare workers and others in high-risk transmission settings [3, 8].

It is essential to recognise that successful implementation of TB-IPC requires sufficient human resources, training, dedicated funding and coordinated actions within the broader IPC framework implemented by the health system. In high-income settings, these measures are typically well-established and supported by ample resources across different hierarchy levels. However, in low-resource settings the capacity to implement structural changes to improve facility safety is often limited, though other solutions can be employed. Innovative approaches in these settings include making effective use of natural ventilation and external waiting rooms, along with streamlined procedures for triage and early detection, and treatment initiation in high-risk environments. The cost of particulate respirators has restricted their availability for target staff, although their production and use have expanded following the COVID-19 pandemic.

## Relevant country experiences

The WHO handbook provides practical examples from various countries on implementing administrative controls, environmental controls and respiratory protection measures [8]. The education of the three actors in this drama (patients, healthcare staff and visitors) is critical for implementation of effective TB-IPC programmes. Nigeria, for example, has played a key role in the development by the US Centers for Disease Control and Prevention of the TB BASICS toolkit, which includes healthcare worker training, baseline and follow-up assessments, and targeted actions to address gaps. Similarly, India has collaborated with international partners to train its staff in engineering aspects of infection control, as part of a broader effort to reduce transmission over recent decades. The handbook also offers standardised education messages that must be tailored to local contexts [8].

Reducing transmission at healthcare facilities is a crucial step in impacting TB epidemiology. The handbook highlights illustrated examples of effective natural ventilation systems in countries, such as Bangladesh, Peru and Viet Nam. Prompt and effective TB treatment can significantly reduce curb TB transmission, even before bacteriological conversion is achieved [3, 8, 9]. The implementation of the FAST strategy in various countries has helped identify many undetected TB cases and exposed the use of inadequate regimens among hospitalised patients [2, 3, 10–14]. For example, in Bangladesh, the FAST strategy uncovered cases of unsuspected TB among hospitalised patients, while in Peru, it improved the diagnosis of drug-resistant TB and sped up treatment initiation in a general hospital setting. In the Russian Federation, the implementation of the FAST strategy in two hospitals resulted in significant reduction in multidrug-resistant (MDR)-TB cases 12 months after implementation.

The WHO handbook also includes a tool to assess the risk of TB transmission in healthcare facilities (annex 2) [8]. Additional resources include the TB BASICS toolkit (box 2.4), guidance on conducting periodic implementation assessments (*e.g.* every 6 months), and a checklist for reviewing the programmatic implementation of TB-IPC (annex 9).

Practical advice is also provided, such as the use of vaneometers (sections 3.11–3.12) for measuring air changes per hour, principles for modifying consulting room organisation (3.14) and cost-effective strategies such as keeping windows open or relocating waiting rooms outdoors (3.17–3.18). These insights are drawn from experiences in countries such as India, Mozambique, Peru, South Africa, Tanzania and Viet Nam.

Healthcare workers are particularly vulnerable to TB infection and disease. The handbook provides guidance on screening them and treating TB infection or disease when necessary (annex 4). It is also important to emphasise the importance of respiratory protection, with the handbook offering advice on respirator fit testing (section 4.1.1). Notably, approximately 50% of participants in a training exercise failed the fit test, indicating that their respirators lacked proper protective efficacy (G.B. Migliori, personal communication).

## Optimising hospitalisation for TB patients

Limiting hospital admissions and stays to the necessary minimum is crucial to reduce exposure to other inpatients, particularly those with different TB strains or those with drug-resistant microbes. Hospitalisation is generally reserved for persons with TB requiring care that cannot be managed on an outpatient basis, or for those who pose a community transmission risk due to their infectiousness. The WHO advocates for ambulatory and decentralised care models, particularly for persons with MDR-TB, over hospital-centric approaches [15]. This shift does not necessarily increase the risk of community transmission, provided appropriate infection control measures are implemented, such as treatment support, respiratory etiquette, using masks and respirators, and enhancing ventilation at home.

Community-based contact investigation offers a valuable opportunity to educate household members on infection control, and to provide TB preventive treatment. Adequate resources are essential to facilitate this shift from hospital- to community-based care models [8, 16].

A 2021 review by the Global Tuberculosis Network (GTN) [17] provided an overview of TB hospital admissions, including during the COVID-19 pandemic, and identified several key findings:

1) The proportion of newly diagnosed TB patients admitted to hospitals ranged between 50% and 100% (47% in Brazil, 57–65% in the USA and Canada, and >80% in the WHO European Region).
2) Approximately 100% of persons with MDR-TB were hospitalised in Europe, with 85% in the previous GTN bedaquiline study [18].
3) During the COVID-19 pandemic, 58% of TB/COVID-19 co-infected patients were hospitalised globally [19–21].

Length of stay for newly diagnosed TB cases varied significantly, from a median of 10 days in the USA to 90 days in the Russian Federation. Persons with MDR-TB typically had a length of stay between 50–100 days in Europe, with some studies reporting up to 180 days for persons with extensively drug-resistant TB.

Key indications for hospitalising persons with TB include [3, 8, 17]:

1) Severe TB disease that cannot managed in outpatient care, such as those with respiratory failure, haemorrhage, pneumothorax or pleural effusion [22–27].
2) Severe pre-existing co-morbidities, such as coma, liver or renal disease, or uncontrolled diabetes.
3) Severe adverse events associated with anti-TB treatment, such as arrhythmias, psychosis or renal failure [28, 29].
4) Infectious patients without bacteriological conversions or those with complicated forms of drug-resistant TB.

Several pre-conditions must be met before persons with TB can be hospitalised, including putting in place the appropriate IPC measures, ensuring the availability of respiratory isolation, training and protocols for effective communication and coordination between healthcare providers, staff, laboratories and peripheral units receiving patients after hospital discharge [3, 17]. However, in some countries regulations require that persons with TB remain hospitalised until bacteriological conversion, which may inadvertently increase bed occupancy and incentivise prolonged stays due to funding structures tied to the number of occupied beds [17].

While clinical improvement of TB continuity of care are the primary criteria for hospital discharge, bacteriological conversion is not necessarily required if infection control measures can be effectively implemented at home [21]. Persons with TB would have typically exposed household members before diagnosis, and transmissibility usually declines rapidly once treatment is initiated [3, 8].

## Monitoring TB-IPC action

**FIGURE 1:**
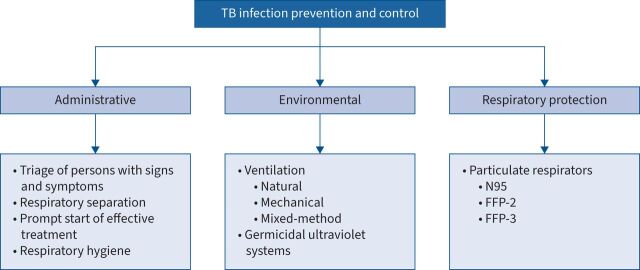
Planning, implementation and evaluation of tuberculosis (TB) infection prevention and control: the core indicators.

Monitoring and evaluation are critical for the success of a TB-IPC programme. In contrast to indicators that focus on patient diagnosis, treatment coverage and outcome, much of TB-IPC monitoring relies on process indicators relating to the enhancement of facilities and changes in behaviour and practices. The following are examples of process indicators suggested in the handbook: is an IPC plan and committee in place? Is triage being performed? Is protective equipment in place? Figure 1 outlines three quantifiable indicators for the routine monitoring of key TB-IPC measures at the national level.

## Conclusion

The WHO operational handbook on TB-IPC outlines a comprehensive three-tier IPC hierarchy applicable in healthcare and congregate settings within the broader framework of national IPC activities. It emphasises the integrations of TB-IPC with broader TB prevention interventions. A multifaceted approach is essential to control TB transmission, starting with early identification, triage and prompt treatment. These measures must be complemented with environmental control, such as well-designed ventilation systems, GUV equipment and particulate respirators, which are only effective when used correctly. Optimising hospitalisation duration and promoting ambulatory and decentralised care is are crucial to reducing transmission in healthcare settings. Monitoring and evaluation complete the framework, focusing on process indicators that address facility enhancements and behaviour change at the national level.

Health services, national TB programmes and private providers must allocate resources to fully implement TB-IPC as a part of a broader prevention agenda, which includes TB preventive treatment, active TB case-finding and addressing upstream determinants of TB. Persisting challenges, especially in high-burden TB and HIV settings, require prioritisation and systemic engagement. Further research is necessary to refine existing strategies and develop new tools, such as a test to gauge infectiousness. Sustained commitment and collaboration are vital to achieving global TB-IPC goals.

## Shareable PDF

10.1183/13993003.00109-2024.Shareable1 This PDF extract can be shared freely online.
Shareable PDF ERJ-00109-2024.Shareable
