# Supplementary material for: Viewpoint on WHO implementation guidance on tuberculosis infection prevention and control
Source: Eur Respir J. 2024 Dec 12;64(6):2400109. doi: 10.1183/13993003.00109-2024 (PMC11635381; doi:10.1183/13993003.00109-2024)
Supplement: Supplementary file 1 [file ERJ-00109-2024.Shareable.pdf]

SHAREABLE PDF

Viewpoint on WHO implementation guidance on tuberculosis infection prevention and control

Onno W. Akkerman [1,2,15], Giovanni Battista Migliori [3,15], Dennis Falzon[4,15], Alberto L. Garcia-Basteiro[5,6,7], Avinash Kanchar[4], Olha Konstantynovska[8], Fusun Oner Eyuboglu [9,10] and Raquel Duarte [11,12,13,14]

[1]University of Groningen, University Medical Center Groningen, Department of Pulmonary Diseases and Tuberculosis, Groningen, The Netherlands. [2]University of Groningen, University Medical Center Groningen, TB Center Beatrixoord, Groningen, The Netherlands. [3]Servizio di Epidemiologia Clinica delle Malattie Respiratorie, Istituti Clinici Scientifici Maugeri IRCCS, Varese, Italy. [4]World Health Organization, Global Tuberculosis Programme, Geneva, Switzerland. [5]Centro de Investigação em Saúde de Manhiça (CISM), Maputo, Moçambique. [6]Instituto de Salud Global de Barcelona (ISGlobal), Hospital Clínic-Universitat de Barcelona, Barcelona, Spain. [7]Centro de Investigación Biomédica en Red de Enfermedades Infecciosas (CIBERINFEC), Barcelona, Spain. [8]V.N. Karazin Kharkiv National University, Department of Infectious Diseases and Clinical Immunology, Kharkiv, Ukraine. [9]FOE Respiratory Clinic, Ankara, Turkey. [10]Baskent University Division of Pulmonary Diseases, Ankara, Turkey. [11]EPIUnit – Instituto de Saúde Pública, Universidade do Porto, Porto, Portugal. [12]Laboratório para a Investigação Integrativa e Translacional em Saúde Populacional (ITR), Porto, Portugal. [13]ICBAS – Instituto de Ciências Biomédicas Abel Salazar, Universidade do Porto, Porto, Portugal. [14]Instituto de Saúde Pública Doutor Ricardo Jorge (INSA Porto), Porto, Portugal. [15]Contributed equally.

Corresponding author: Onno W. Akkerman (o.w.akkerman@umcg.nl)

Shareable abstract (@ERSpublications)
This viewpoint describes key aspects for TB infection prevention and control, and also covers areas of paramount interest for clinicians, such as when to admit or discharge infectious patients and presenting relevant country examples https://bit.ly/3YcyJxj

Cite this article as: Akkerman OW, Migliori GB, Falzon D, *et al*. Viewpoint on WHO implementation guidance on tuberculosis infection prevention and control. *Eur Respir J* 2024; 64: 2400109 [DOI: 10.1183/13993003.00109-2024].

This extracted version can be shared freely online.

Check for updates

Copyright ©The authors 2024.

This version is distributed under the terms of the Creative Commons Attribution Licence 4.0.

Received: 16 Jan 2024
Accepted: 15 Oct 2024

Transmission continues to drive the tuberculosis (TB) and drug-resistant TB epidemics, making infection control an essential component for public health agencies worldwide [1–3]. Transmission of TB is complex, influenced by factors linked to patient behaviour, the form of disease, the exposed individual, the microbe and the environment [3–6]. Each year, more than 10 million people develop TB, more than 80% of whom have pulmonary disease and more than 60% are bacteriologically positive [1]. Determining infectiousness is difficult in the absence of a reliable biomarker for point-of-care triage of TB patients. While sputum bacteriology status correlates with infectiousness, its accuracy is limited, especially after initiating effective TB treatment [3, 7].
